# A novice to expert analysis of skill development in birth doulas

**DOI:** 10.3389/fgwh.2025.1604410

**Published:** 2026-01-16

**Authors:** Amy Louise Gilliland

**Affiliations:** School of Human Ecology, University of Wisconsin-Madison, Madison, WI, United States

**Keywords:** doula, labor support, intuition, novice to expert, birth, maltreatment, longitudinal, grounded theory

## Abstract

**Introduction:**

This study applies the Benner interpretation of the Dreyfus Model of Skill Acquisition to birth doulas.

**Methods:**

Sixty-five doulas participated in open-ended interviews in five waves between 2002 and 2022. Constructivist grounded theory methods were used to collect and analyze the data. Participants attended over 25 births, spoke English fluently, and did not utilize any medical skills. The doulas ranged in age from 22 to 65 and practiced in a variety of areas and settings in the United States, Canada, and the Netherlands.

**Results:**

The Benner model was relevant. Birth doulas grow similarly to nurses from novice to expert, including the development of intuition. However, the skill set is different. As they improved in skill acquisition, birth doulas showed advancement in information processing; confidence; decision-making; communication; self-awareness; client and staff relationships; professional detachment; definition of an ideal birth; management of witnessing medical maltreatment and feelings of overwhelm; the ability to read client cues; anticipation of labor events and staff responses; managing the challenges of a professional doula lifestyle; sense of identity, the maturation of expert intuition; and awareness of when they had power to influence a situation. Swiftness in development depended on the variety of birth experiences and locations; the doula's ability to reflect and find meaning; and life and career background.

**Conclusion:**

Birth doula work is more complex and multifaceted than previously thought and requires growth in specific skill sets to be successful. Effective birth doula work requires sophisticated emotion management, analytical and communication skills, in addition to labor support skills. Public perception that anyone can be a doula is erroneous. It is a separate profession from obstetrical nursing, although some skills may overlap. Rather than continually training new people, programs could concentrate on removing the challenges to continuing birth doula work. Doula programs should address the challenges of each stage, thus encouraging greater expertise and retention and growth of an experienced workforce.

## Introduction

Throughout history, women have been assisting other women during labor. Emerging in the early 1980s, the birth doula is the most recent incarnation (1–3). Doula care has changed in the last 25 years from being viewed as a luxury service for affluent people to a public health strategy for changing racial inequities in birth outcomes (4–7).

The birth doula possesses skill sets in emotional support (8), physical support (9–11), information (12), and advocacy (13, 14), and as a source of pain prevention and relief (15). However, no peer-reviewed article has examined how the doula develops these skills over time. Among skill acquisition models, the Dreyfus Model of Skill Acquisition as interpreted by Benner (16, 17) provided the best fit for understanding this process.

To Dreyfus and Dreyfus (37), the movement from novice to expert reflects changes in three aspects of performance. First, the professional's working paradigm shifts from reliance on abstract principles to concrete past experiences. Second, the professional shifts from seeing situations as discrete unrelated parts to seeing situations as part of a whole. Third, the professional's position shifts from detached observer to involved performer (38). Benner extended these concepts through five stages of nursing skill acquisition: novice, advanced beginner, competent, proficient, and expert.^1^

A recent study followed the internal processes of nurses as they became familiar with the philosophy of patient-involved decision-making and used the tools and patient activities (19). Their technique supported the patient's development of problem-solving and self-management skills, similar to the birth doula's role. At the novice level, nurses failed to understand empowerment. They were occupied by learning the techniques. At the advanced beginner level, nurses appreciated the tools as enabling deeper conversations. They focused on the value of the communication detached from the value of the total philosophy. At the third level, nurses now appreciated the patient's perspective. Proficient nurses began to see the activity sheet as a tool for the patient's independence. Expert-level nurses were creatively collaborating with patients. Key skills in their development were mastering unpredictability, complexity, meaningfulness of the work, and self-reflection and awareness. These themes offer clues when analyzing birth doula narratives.

Benner showed that nurses developed clinical competence using the Dreyfus Model of Skill Development, and application of this theory has contributed to setting professional standards (20, 21). Our first question is whether the Benner interpretation of the Dreyfus Model of Skill Development is applicable to birth doulas.

Nurses learn by doing the tasks of nursing and improve their skills through active reflection upon their experiences (22). Patient interaction has to be balanced with the cognitive load and ethical responsibilities of their job. The same can be said of birth doulas. In exploring skill progression models, the Benner interpretation has the most relevant literature. Further parallels emerge between nurses and doulas. As Evans wrote (23):

“Nursing, like other disciplines, is defined by its societal obligations, goals, values, ethical framework, knowledge base, discipline-defining theories and the skills it uses to meet the health needs of the people it serves. It is not only the skills, but it is the perspective in which the skills are performed that defines the work of nursing.”

It is that shift in interpretation of context that defines each stage of growth for both the nurse and the birth doula (8). What they are thinking makes the difference, even though the action (i.e., counterpressure) might look the same. Both nursing and birth doulaing^2^ have been subject to the same erroneous assumptions. Birth doulaing is only recently being recognized as a valuable strategy in combating racial inequities (24–26) and an autonomous paraprofessional role (27). In a study furthering the Benner model, Evans stated: “…the perception by nurses, other healthcare disciplines, employers, and the public is that nursing knowledge is merely a subset of medical knowledge. This leads to the view that autonomous decision-making is minimal and the expectation that nurses deferred decision-making to physicians or administrators.”

Likewise, doula knowledge is assumed to be a subset of nurse knowledge. By outsiders, doula support is often seen as a list of labor support tasks rather than an identity and lifelong pursuit (28, 29). This thinking renders additional skill sets in advocacy, communication, and emotional intelligence to be invisible (30–32). Birth doulas make ethical decisions autonomously and frequently. These decisions are often contextual (14, 33), which also mirrors the nursing role.

The purpose of this study is to describe how birth doulas develop their skills and professional perspectives over time and to determine whether Benner's interpretation of the Dreyfus Model of Skill Acquisition provides a meaningful framework for understanding the developmental progression of doula practice.

## Materials and methods

The primary research question was, “What is effective labor support by doulas?” At the time of the first process of data collection, no prior inquiry had examined the doula's role or activities in depth. An open-ended question was selected to reveal the richest possible data about the doula's inner processes and perceptions. Because the relationships and social processes of doulaing are complex and contextually shaped, a grounded theory approach was used (18, 34). As the study evolved, it became clear that doulas at different levels of experience held different perspectives and used their skill sets differently. Various models of skill acquisition were explored, and the Benner interpretation of the Dreyfus model provided the best conceptual fit for the data.

### Design

Open sampling in a grounded theory study requires that the selection of interviewees be relevant to the research question (18, 35). Approval for the study was received from the University of Wisconsin-Madison Human Subjects Committee from 2002 through 2022. Participants received a $10 gift card (2002, 2005, 2010) or $20 gift card (2017, 2020) as a thank you gift.

### Participant and inclusion criteria

Recruitment methods varied with the technologies available at the time and are listed in Table 2. Doulas were recruited through email announcements and social media, as well as word of mouth. Interested doulas contacted the researcher, were informed about the study, signed a consent form, and were interviewed (see Table 1 for participant description and Table 2 for sample recruitment.) Doulas needed to have attended over 25 births, to speak English fluently, and not use clinical skills in any capacity. (In 2002, it was unknown that 25 births would end up omitting novice doulas from the sample).

**Table 1:** Participant data.

Category	Details
Total number of doulas	65
Interview time span	Twenty years, in five waves
Geographic diversity	Twenty-six US States, three Canadian Provinces, and one the Netherlands
Age range	22–65 years
Practice locations	Large cities, small cities, and rural areas
Birth locations	Home, freestanding birth centers, and hospitals
Economic and social diversity	Varied income levels, religious faiths, and economic backgrounds
Racial and ethnic composition	Fifty-five Culturally white (84.5%), four Black (6%), three Latina (5%), two Asian (3%), and one Arab-American (1.5%)
Immigrant doulas	Five white doulas were adult immigrants to the United States.
Doula program employment	Twenty-seven employed by hospital or birth center doula programs
Community-based doulas	Ten were community-based with long-term client relationships

**Table 2:** Sample recruitment.

Year (wave)	Locations	Sample size	Interview method	Recruitment method	Characteristics
2002 (1)	International Conference Rural Florida Rural Long Island, NY New York, NY Chicago, and IL suburbs	30	In person, audio-recorded	Doula email networks Conference program Word of mouth	Open sampling Theoretical sampling: geography, client type, and hospital policies
2005 (2)	Columbia, South Carolina	6	In person, audio-recorded	Hospital-based doula program	Theoretical sampling: hospital-based doula
2010 (3)	Minneapolis, MN Milwaukee, WI	8	In person, audio-recorded	Email Facebook Posts	Theoretical sampling: hospital-based doula novice doula
2017 (4)	International conference	7	In person, audio-recorded	Doula Social Media Networks Conference organizer	Theoretical sampling: doula agency Owners 100+ births
2020–2021 (5)	Video call	14	Video call and video- and audio-recorded	Social media promotion	Theoretical sampling: hospital-based doulas

During each recruitment wave, efforts were made to ensure diversity among doulas. In 2002, the first wave of 30 doulas sought out doulas from different regions of the country and in different job types. In 2005, the focus was on hospital-based doulas who had attended hundreds of births without ever meeting a client before labor. This enabled labor support to be isolated from other doula functions. In 2010, the sample was expanded to include hospital-based doulas from four different programs in two different cities. This could serve to validate emerging concepts and ensure that they were not specific to one program. By 2017, training approaches and practice models for doulas had diversified, and doula agency owners were contacted and interviewed. This group had large numbers of births, a variety of doula experiences over time, and had become leaders. In 2020, I returned to hospital-based doulas to further refine skill development in the birth doula role. However, most had had a few clients they saw prenatally. In the last two waves, purposeful efforts were made to increase the racial diversity of the sample to reflect the changing workforce and to be more informed on how race or ethnicity made a difference.

### Participant characteristics

A total of 65 birth doulas participated in the study. They ranged in age from 22 to 65 years and were recruited across 26 US states, three Canadian provinces, and the Netherlands. The majority identified as white (*n* = 55; 84.5%), with others identifying as Black (*n* = 4; 6%), Latina (*n* = 3; 5%), Asian (*n* = 2; 3%), and Arab-American (*n* = 1; 1.5%). Five participants were white immigrant doulas who had moved to the United States as adults. Participants represented a range of socioeconomic and religious backgrounds and practiced in a range of settings, including large cities, small towns, and rural areas. Births were supported in homes, freestanding birth centers, and hospitals.

### Collection period

Data were collected over a 20-year time period in five waves.

## Data collection and analysis

Interviews were conducted individually by the author and lasted 90 minutes to 2 hours. Fifty-one were in person; the last 14 were conducted by video call. All were audiotaped. For consistency during analysis, only the audio was analyzed from the video calls. Transcripts were an average of 28 single line pages, with a range of 17–52 pages.

Questions were consistent over the 20-year time period. Doulas were asked, “Please share a time when you felt your doula support was really effective. You can decide what effective means, start wherever you wish, and include as many details as you want.” Doulas were encouraged to share more than one story. Follow-up questions used a hermeneutic approach, meaning that the goal was for the participant to focus more deeply on their actions and provide their own contexts for why this particular time was considered effective and why it was meaningful to them. Time was spent deeply examining the stories they chose to share. Another consistent question was to list the skills or qualities they felt were important for a new doula to develop. Follow-up questions used topics or phrases organically presented by the participant. Typical topics were terminology, clients, providers, nurses, partners, and the joys and challenges of doula work.

Constructivist grounded theory was used to explore relationships, skill development, and interactions among birth doulas (35). This approach allowed for flexible, iterative data collection, emphasizing the coconstruction of meaning between researcher and participants in the interview process. Using the constant comparative method (35) allowed patterns to emerge from the data. In order to explore developing concepts, theoretical sampling guided the waves of participant selection. For example, waves two and three had doulas who were exclusively hospital-based, and wave four was made up of doulas who were highly experienced and primarily agency owners.

Efforts were made to diversify the sample racially in the 2020/2021 interviews of hospital-based doulas. Black doula organizations and groups were contacted, and individuals made appeals within the organizations for people to participate. However, efforts were not successful enough to actually make the sample diverse. Restrictive factors were the general racialized atmosphere in the United States at the time (fall 2020 through spring 2021); the researcher was white and unknown to them; compensation for their time was inadequate due to IRB agreements; and the small number of hospital-based doula programs were staffed with Black doulas. There was also an influential discussion on social media about participating in research that did not originate with the Black community.

Thus, 65 doulas participated in interviews between 2002 and 2022. The interviews were transcribed, verified, and coded line by line, ensuring rich, nuanced interpretations. While labor-intensive, this allowed for multiple codes to be applied to each segment, capturing different meanings. All interviews underwent this coding process to ensure consistency and reliability. No doula interviews were excluded from analysis.

Grounded theory methodology holds that a detailed in-depth analysis of small units of data is necessary in order to extract significant levels of meaning (18). Out of this in-depth deconstruction of the transcribed interviews, the grounded theory researcher is able to reconstruct a model that fits the data. Data were organized using a computer program, but no AI or coding software tools were used, ensuring that the analysis remained researcher-driven. Focused and theoretical coding encouraged micro-level data analysis (35), allowing the creation of a theory grounded in the experiences of the doulas. Memo writing helped document reflections and clarify emerging themes (36).

Differences in perspective emerged almost immediately. Doulas who had attended over 500 births had different insights from those who had been to only 30. Different models of skill acquisition were considered and the Benner framework of the Dreyfus model provided a worthwhile lens to view the data. However, this did not change the interview questions or process. Data were coded and analyzed after each wave. Upon comparing coding and conclusions from all waves, it was found that there were no differences, affirming similar skill development decades apart. The doulas tended to have similar epiphanies. The perspective of novice doulas was provided by more experienced doulas reflecting on their earlier state of mind. They were also asked, “What are the skills or qualities do new doulas need to possess to be successful?” The literature confirms that new learners are often uninformed about their own thinking processes (37, 38).

### Rigor and reflexivity

To minimize the impact of bias, multiple procedures were followed and are listed in Table 3. Some procedures changed over time. Rather than member checking with doulas who had been interviewed, concepts were cross-validated by discussion groups of doulas not in the original study. This was done for two reasons. First, in 2002–2006, member checking with the original group was a daunting task, as the sample of 30 was spread across the eastern United States and Canada. As a graduate student, I was discouraged from sharing research documents on paper with participants. Because of the technology at the time, that was the only option available. Second, since the transferability and generalizability of the concepts was paramount, it was considered appropriate to check the data with a discussion group of doulas who were not interviewed. If the data were valid, they would concur with the findings. Concepts were viewed as reflective of the doula's experience and skill levels by the discussion groups. To ensure that earlier data (2002–2005) were not outdated, excerpts were shared with contemporary doulas. They were identified as relevant and could not be distinguished from more recent excerpts. Although the data were not used here, a sample of 30 parents who received doula support expressed viewpoints consistent with those of the doulas. All participants were assigned pseudonyms.

**Table 3:** Reflexivity and rigor: procedures followed to reduce bias in the study.

Procedure	Description
Diverse interviews	Interviews were conducted with a diverse group of doulas over 20 years, representing various ages, locations, and backgrounds.
Detailed notes and memos	Rich descriptions and detailed notes/memos were taken to accumulate strong evidence for each finding.
Peer debriefing	Periodic peer debriefing sessions were held with advanced degree holders who were doulas to uncover hidden biases.
Negative case analysis	Negative case analysis was used to set aside concepts that did not fit with existing ideas until new concepts emerged.
Purposeful sampling	Purposeful sampling ensured representation across ages, races, religions, geographic regions, training types, organizations, and philosophies of care.
Cross-validation	Concepts were cross-validated through discussion groups of doulas not in the original study.
Contemporary validation	Excerpts from 2002 to 2003 and 2005 data were shared with contemporary doulas for validation of relevance.
Parent feedback	A sample of 20 parents who received doula support expressed viewpoints that agreed with the doulas’ perspectives.
Pseudonyms used	All names in the study were pseudonyms to ensure confidentiality

### Ethical considerations

Trustworthiness and the researcher's relationship to the topic are key. I had been a doula for 15 years in 2002 and attended births until 2015. A bidirectional relationship developed between data analysis and professional growth. My skill development progressed rapidly as I applied their suggestions with my own clients and to training new doulas. This directly impacted my ability to be an instrument of analysis and interpretation because my own doula skills improved. This process of insight and application deepened my understanding of doula skill growth, including intuition, which is often valued in qualitative methods (39, 40).

## Results

Across the 65 doulas interviewed, skill development followed a clear progression from novice to expert. Key differentiating factors included (a) the number and variety of births attended, (b) internal reflection processes, and (c) management of relationships in clinical contexts. Novice doulas felt that the situation happened to them somewhat randomly. They could not discern what information was important and what was not, which made the situation feel unpredictable. They relied on the concept of an ideal birth outcome. They took actions personally as a reflection on their performance. A sense of disconnection persisted through the advanced beginner stage along with the growing awareness that laboring patients could experience harm. They often felt overwhelmed and looked outside themselves for validation. They became disillusioned with any rules they thought would make birth predictable. Instead, doulas at this stage learned to focus on meeting the client's needs and not taking things personally.

Seasoned doulas balanced the need to look externally to judge their success with a growing sense of internal validation. They learned how to prioritize labor support activities. Cues seemed connected and no longer random. They could anticipate events and communicated more confidently and empathetically. In the proficient stage, doulas were more self-aware and attentive to their own self-care. Birth had become known yet remained unpredictable. Doulas were confident that they would be able to support their clients no matter what. They could influence a situation through their caring presence. In the expert stage, birth doulas evaluated the success of their support solely through their own lens—did the client get their needs met? External factors no longer mattered. They relied on their embodied intuition in meeting clients' needs and in their decision-making—often without understanding why. Rather than seeking outside for answers, expert doulas actively constructed knowledge based on their own experience. Knowledge had become embodied.

### Novice doula

Birth doulas hold the fundamental belief that clients define their own ideal birth. Their role is to assist the client to discover and support them in their vision. At the novice stage, this ideal birth is often outcome-oriented. Most people enter the field with this mental mindset. As Bree said, “Earlier in my career, I probably nudged people to decline or accept things based on my own ideas.” Scout outlined her mindset at the time. “My first few clients didn't want an epidural, and one of them ended up getting one, and I felt like a failure, like I had not done enough to prevent this medication … I was sad for her and frustrated with myself.”

The newly trained doulas in Doris' program said they would be ready once they had attended a birth with someone else. Doris laughed, “It doesn't work like that. It's important to have the willingness to go into that territory … If everything you do is based on doing it before, you’re no good to her. Because every labor is a new one.”

Several doulas felt they needed to justify their presence. Bree shared, “Eight years ago I would have done everything I could to get into the place [the father] was in, to take over that role, because I would have felt like I needed to be doing that.” Riko says, “I've seen this with a lot of new doulas, you're not directing or calling the shots. That is what makes doctors and nurses very upset. You're not the birth planner … You are just there to support and encourage.”

Doulas quickly realized that there were a few rules that often brought overwhelm and uncertainty. With each birth they discovered how much they did not know. Part of transcending unpredictability and uncertainty is moving forward despite feeling unprepared. Scout spoke for most participants: “I was really starting to realize very early,” “Whoa, none of this is under my control, and there's a bigger bag of stuff out there than I realized.”

### Advanced beginner doula

Novices rely on rules and guidelines learned in their training. In the advanced beginner stage, their performance increases in terms of reliability. Certain aspects of hospital routines or labors become familiar. Advanced beginners start to differentiate situations but still have difficulty distinguishing important cues (18). In unpredictable situations, they may not know where to put their attention.

Advanced beginner doulas have a shift in cognition described as chunking (41). Chunking is the process by which larger units of behavior or cognition come to be seen holistically as a single thought or action. The novice sees letters, and the advanced beginner sees syllables. Their concept of an ideal birth changes. Camille shares, “Even though I intellectually understood that it's not about my right kind of birth, it took a while to have enough experiences where you really see, ‘Wow. This person so benefited from that epidural,’ or, ‘Their labor really got better after they had that IV and were hydrated.’”

One of the markers of birth doulas who transcended this stage was confronting and discarding their own biases. They had to detach their clients' behavior from being anything personal. Doulas had to see birth as something that happened while they were accompanying their clients and not as something that had a universal ideal goal. Scout says, “I had to learn that birth wasn't about me. It's not about what I do for the family, it's about how the family feels.”

At this stage, doulas have experienced differences in staff behavior and concerns. They are surprised at how often the mother or baby's needs are not prioritized. The hospital system can be bewildering and uncaring. Sometimes, doulas are uncertain whether they are witnessing maltreatment.

Thalia: They'll let you slowly suck on ice chips. Then if the ice chips melt and you drink it, you get yelled at. That's unbelievable.

Naomi: I'd forgot to ask her, “Do you want the baby right away or not?” So, baby goes over to the warmer. I mentioned to the nurse, “She’d really like to get her baby right away.” Nurse was busy doing all of her stuff. “Well, I’m really busy right now.” So, I pushed it. I said, “Well, you know, it is *her* baby.” Afterwards I realized how out of line that was, but I think it's so criminal. She blew up. “That was really out of line for you to say. I’ve got things to do and I’ve got to do it my way.”

Lydia: If you begin to take it personally…it's so easy to throw your hands up and walk away. I can't tell you the number of births afterwards I was like, “Why am I doing this? Why do I stay up all night, stay away from my family, treat my body badly, as you know, only to see her suffer?” The reality is, “Did she have a better birth because I was there? Probably. But because I take my job very personally, it hurts when it doesn't go right.”

### Seasoned doula

The seasoned doula actively retrieves knowledge on aspects familiar to them but still looks outside for context, clues, and validation when uncertain. At this stage, feeling overwhelmed lessens. Overwhelm is the emotional feeling of the flooding of a person's neurological senses. They are coping with multiple sensory inputs simultaneously and do not know where to put their attention first. Seasoned doulas can prioritize and integrate information; they have now developed a mental schema to sort out what is important and manage their own responses. Confidence is key, as Riley states, “I can use things in that birth room in ways that you can't even imagine. But if I don't have the confidence to do it, all that information in the world doesn't help me.” Lani's confidence shows up in a different way. “I go to a birth with my birth ball, a hot sock, and some oil. And that's it. My heart and my hands and my voice are all I bring. Tea, my glasses … stuff for me. We just don't need a lot of stuff.”

The seasoned doula focuses on their role, what they do know and what they can influence in a situation. Uneven treatment of mothers and other people in labor is no longer new or novel. Some doulas reported they witnessed maltreatment of their client at every birth. Their mental process focuses on, “What is the priority here? What do my client and family need most? What can I do?” Serena says, “You’re there with a woman who happens to be part of a public health clinic system and you see her being treated like a guinea pig … You’re trying to keep her spirits and morale up and show some respect.” In a contrasting situation, Scout explained, “Because I had a relationship with the nurses, they felt comfortable asking me,” “Can you help us better care for this patient? We want her to have a good experience.”

At this stage, the doula is still evaluating their performance according to external metrics—their cesarean or epidural rate, or how many intended VBACs were successful. There is a burgeoning sense of relying on a more holistic sense of success: “Was the client 100% supported? Were the client's and family's needs met?” This shift is necessary to move on to the next stage. Bree illustrates her thinking. “Births weren't happening the way that I wanted them to happen. When I took it away from being about me, or what births should be like, or confirming my biases, I think that very much contributed to my sustainability.”

### Proficient doula

At the proficient level, birth doulas have begun the process of reflection in action: “a particular kind of mindfulness which involves an intense concentration on the task at hand” (37). Reflexive thinking relates all actions to the patient and situation without conscious thought. This intense focus is possible because the landscape of birth has become familiar to them. Ashley shares, “That's what was strange, I called every shot ten minutes, fifteen minutes before it happened. I’d explain it to them, and I’d say, “Look this might be what's coming next.”

Proficient birth doulas are very clear about their role and know where they have power and influence, even though none of the important decisions are theirs. As Scout outlines, “None of my perspective matters. I don't have to go home with the consequences … You have to make these really hard decisions sometimes, and your care provider has to make really hard decisions sometimes. I'm here to facilitate communication more than anything.”

In familiar situations, proficient doulas begin to act reflexively. When faced with novelty, it may take the doula a while to process and arrive at the best approach. Minal makes it clear. “You've got to learn when to give them some space … when a doctor has just come in and said, ‘I want to put Cesarean birth on the table’, maybe it's time for you to go get a coffee. You don't have to be right there in their face, ‘What do you think?’ Go. Give them a minute. Let them cry.’”

Proficient doulas have become savvy about their relationships with medical staff. Their biggest leverage is in how they set it up when the staff arrive.

Scout: “I make a point to introduce myself, to recognize…that this is their home, and I'm a guest. Even if I know exactly where the towels are, if the nurse is in the room, I always check in with her, ‘Hey, do you mind if I do this?’ Because that's my way of recognizing their authority in the room.”

They have established a separation between the outcomes of birth, the client's feelings, and their own performance. This is a boundary that did not exist in the previous stages.

Scout: “I had a client who seemed very unhappy that she had an unplanned cesarean. I’m confident that there's nothing that I could have done. There's nothing her provider could have done. It is what it is, and I’m at an emotionally healthy enough place where I can take that. She was so unhappy she declined the postpartum visit. And then a year later out of the blue, I got a text from her. Her baby fell out of the stroller or something. I thought, well clearly it wasn't about me and she knows it because she wouldn't have reached out to me.”

By the time they reached this stage, doulas had found ways to cope with the treatment they witnessed in hospitals. Some moved away from doula work or hospital births. Tracy shared, “My supervisor at the hospital doulas at home births because that's the only way she can survive.” Lani says, “I’m doing less hospital births and teaching more classes. People need to learn how to just say ‘no’ and take responsibility for their own experiences. I’ve got to teach people how to do that.” Sonia stated, “In the city the more experienced doulas make boundaries: what hospitals they’ll go to, what doctors’ practices they’ll work with. There's a lot of doulas who only do birth center or home births, because they don't want to deal with anything else.”

Interestingly, several of the proficient doulas mentioned how they had broken through their internal taboos about touch. In every case, the birth doula followed their inner prompting or “intuition” rather than their social conditioning.

Lydia: [When we've never met] I don't usually touch them right away, but in this case she was in the middle of a strong contraction, and I knelt so that we were eye-level, and just picked up her hand—I usually would ask—and did it. She told me after, several times, the second she felt my hand, she knew it was going to be okay.

Char: Permission to touch moms. That was really hard for me. Now I touch them everywhere. I would say the first couple years I was really uncomfortable with that…It was, “Well, this is professional, and this is what I'm supposed to do. I'll rub her back. But I won't dare climb in bed with her, or hold her, or hold her face and talk to her.” It took me a long time to be comfortable with that.

### Expert doula

The expert doula claims not to think about labor support, instead they act. As Colleen said, “Sometimes there are things that I think about doing and yet I don't do them. I have to assume it's because I just don't feel that that's the right thing for her.” Alison now enters the labor room without any plan. You have to look and see what they’re doing. They’re going to do something instinctively. If they feel safe that will help them get there…That's why when people say, “What do you do in labor for pain coping?” I’m just like, “Oh dear, I don't know. Whatever pops into my head at the moment.”

Expert doulas freely talked about the negative aspects of their work, often with a sense of humor. They discussed the challenges along with successful solutions. In previous stages, these struggles may have felt overwhelming, but now they are part of the job. Bree shares, “I feel like I can anticipate how the birth will go and when it doesn't, I'm frustrated and I kick myself. I'm kind of jealous of new doulas because they're so excited and they love this work, and they just can't wait for the call.”

The Benner model emphasizes the importance of intuition in the expert stage. Intuition was a robust code with contributions from 12 participants. The expert may not even understand how it is that they know what to do but they have gotten over any impediments to following these inner prompts. Expert doulas provided their own explanations and descriptions of these experiences.

Joanne: If I trust my intuition I’m always right. If I don't trust it I'm always wrong. If I think, “Oh, I can hang out in front another ten minutes, she won't have that baby”, I miss the birth. I've been trying for the last three years to analyze what my intuition told me. Was it the look that she gave me? Was it the eye shift? Was it a mannerism that came out? Now every time I have an intuitive feeling about something, I'll step back and think, “What did she do that made me think that way?”

Expert doulas figure out what cues to pay attention to. Their cognitive intuition is so efficient that they often act without thinking, yet it is often the best response. The expert practitioner no longer follows rules but actively constructs knowledge (37). Their actions have become embodied (23, 38, 42, 43). Mia's words echo those of many expert doulas: “I have a very strong intuitive side. The more you get to know women in labor the more you develop that … I trust my instincts to know what to do.”

The expert doula's approach to client relationships has shifted. Their approach is much more relaxed. They seem much less driven to share information than doulas in the earlier stages and more interested in listening. They are more invested in empowering the client than in being the person who gives the answers.

Iris: “Other doulas will say, “All mothers think that.” They’ll negate that. I listen to everything they say and I make it important.”

Tulip: I said, “Ask her how many successful VBACs she's had.” The doctor said she didn't know offhand what her stats were, and just kind of hemmed and hawed and changed the subject. Then [my client] came back and said, “You know something about her I don't, don't you?” I said, “She's never had a VBAC that I know of.” She said, “Can you recommend another doctor?” I said, “Yes.”

Expert doulas mentioned the importance of self-care more often than any other group. Years of doing the hard physical work of labor support and healing from injuries led them to be more cautious. Eve says, “Just recently I thought, Why don't I raise the bed and look at her face-to-face? Why am I bending over? This is dumb. I know better.”

Brione: It's okay for you to take a break and go get some food. It's okay for you to call in back up if you need it. As a new doula, I was just like I have to be awake and I have to be doing all of these things all the time. Which ultimately leads to not the best decisions or the best support. Over the years, I got better at finding, “Where is the chair in the room? Where can I sit? Where can I go to save my back?” And really being kind to myself.

One of the hallmarks of the expert stage was how often birth doulas mentioned “letting go.” Letting go is a process that is ongoing; it is not a single act. It required going through the first four developmental stages to reach this sense of freedom and confidence in all aspects of doula work. Almost all expert doulas mentioned this in one way or another and often without elaborate explanations. Brione says, “The knowledge that you don't make anything happen. The only thing that you can do is inform, and support, and letting all of the rest of it go.” Char adds, “Now I'm kind of just going with the flow. I almost felt like I had to control it back then. Where now I know I'm not in control of anything. I'm along for the ride as well.”

Brione summarizes another expert skill: “To be able to see somebody's discomfort and be okay with it and not try to fix it is not something everybody can do. I think it's something that the doulas learn over time. I think a big part of us early on do want to fix it and part of the lasting aspect of it is having to learn that you can't fix it and that it's okay.”

### Validity of the Dreyfus model to birth doulas

Birth doulas demonstrate the three components of the Dreyfus model: moving from abstract principles to concrete past experiences; from seeing situations as discrete parts to seeing them as part of a whole; and from being a detached observer to an involved performer. The patterns of development of doulas align with Dreyfus' model and Benner's nursing framework, including shifts from rules to intuition, fragmented cues to holistic schemas, and detached to embodied practice.

### Number of births attended and stage attainment

**Figure 1:**
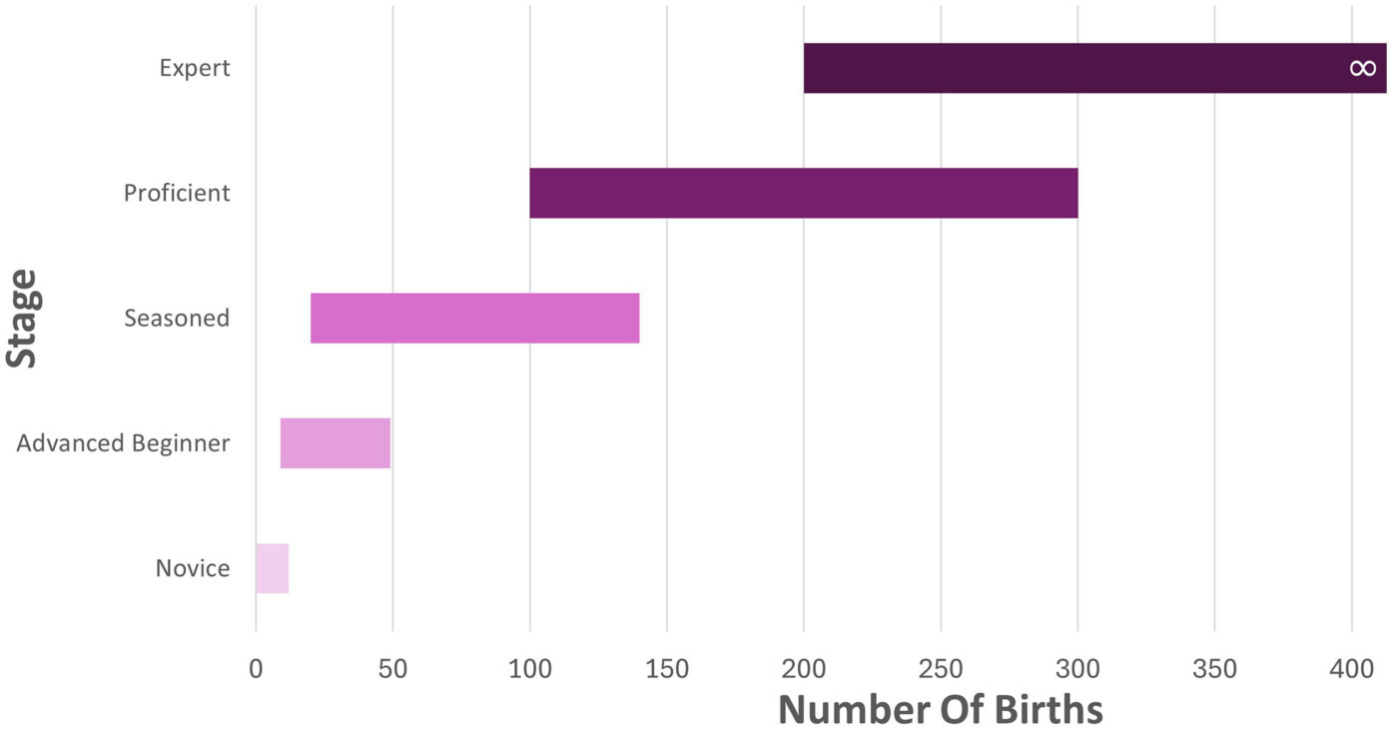
Trends in the number of births and stage attainment.

Trends in the number of births are shown in Figure 1. First, with each phase, the number of births required to progress was significantly higher. Two, being challenged by a variety of support experiences mattered. Doulas who served homogeneous communities or one hospital progressed more slowly out of the advanced beginner phase. Variety stimulated the internal processes of reflection and finding meaning, which were integral parts of skill acquisition for birth doulas. Three, additional career and life experiences likely contributed to how quickly birth doulas progressed through the stages. For example, typically doulas did not reach the expert stage until several hundred births. However, one participant, retired after a 30-year career in counseling, expressed a proficient to expert perspective after supporting 85 families (Table 4). Therefore, the number of births attended can be used only as a reflection of general progress at this time.

**Table 4:** Trends in the number of births and stage attainment.

Stage	Typical minimum	Typical maximum	Range
Novice	0	8	0–12
Advanced beginner	9	25	9–40
Seasoned	30	100	20–120
Proficient	100	200	100–200
Expert	200	none	(85*)200 – no maximum

* Participant with expert themes with only 85 births.

### Changes over 20 years

While healthcare systems, policies, trends, and cultural context changed over the 20-year time period, labor support itself as described by participants did not. As the interviews were analyzed, they were compared across participants with similar and contrasting characteristics. None of the extraneous factors that changed over the 20-year period had a discoverable bearing on the doula's professional development. It was similar no matter where or when they were. Documented changes included but were not limited to the following: baseline health in clients; eligibility for homebirth; need for education; healthcare provider access; education access; access to birth control and abortion; availability of water immersion (i.e., pool, tub) for labor and birth; pain medication practices; policy initiatives in facilities; provider practice style; protocols for cardiovascular conditions; new technologies; cesarean birth rates; COVID-19; hospital obstetric unit closures; public health strategies; delayed cord clamping; infant “skin to skin” policies; and the value placed on the doula. While these factors certainly influenced how birth is managed, what doulas experienced, and the availability of clients, it did not change how the doulas developed their skills over time.

### Maltreatment

Doulas in the fifth wave in 2020/2021 were more likely to identify maltreatment compared with the first wave in 2002. Out of 30 doulas in 2002, only a third mentioned maltreatment, although all doulas described incidents that could be classified as such (44). In contrast, all 14 doulas interviewed in 2020/2021 identified patient maltreatment consistent with Vedam's definition (44), including vaginal examinations conducted without permission; loss of autonomy; being shouted at, scolded, or threatened; and being ignored, refused care, or receiving no response to requests for help. In the immediate postpartum, mothers sometimes did not hold their newborns despite repeated requests, often because of power struggles with nursing staff. The incidents described in 2002, however, were more egregious and included overt coercion and manipulation. Doulas recounted providers performing artificial rupture of membranes without consent during a biophysical profile visit, lying to clients about medication effects, skewing test result interpretations to pressure clients into cesarean births, and chastising a client for hiring a doula. In sum, doulas reported witnessing maltreatment throughout the study period; however, the forms it took were described differently over time.

### Summary

Salient factors relevant to each stage of birth doula development are summarized in Table 5. What they considered to be an ideal birth changed over time as did their sense of purpose. In the first two stages, their purpose was outcome-oriented; success was defined by whether goals were met. In the seasoned stage, this emphasis shifted to whether the client's needs were met in the moment. By the time they reached the proficient and expert stages, doulas had learned to trust and rely on themselves, and whatever they did in the moment constituted the best support for their clients. Outcomes were much less important. As a doula's sense of identity shifted, so did the language they used to describe themselves. As they grew in the level of experience, participants claimed the title of doula as their professional identity. The novice might say they were going to births as a doula but not yet internalize the role. Advanced beginners might see themselves as doulas but not with confidence. By the time they reached the seasoned stage, an appreciation for the professional skills they had acquired added an additional layer to their identity. By the time they reached the proficient stage, their doula perspective had permeated every aspect of their lives. At the expert stage, doulas were applying their mastery in other professional roles or pursuits.

**Table 5:** Birth doula themes and expressed sense of purpose and identity statements at each stage.

Stage	Typical themes, quotations, and purpose and identity statements
Novice	Theme: Tends to follow rules; wants to know what the “rules” are so they can say and do the right thing. Needs to find their niche in the labor room. “At the time I felt I had to justify why I was there.” Purpose: Help their clients to act to get their version of an ideal birth. Identity statement: “I’m going to births as a doula.”
Advanced beginner	Theme: Begins to realize that rules don't always apply. There is room for flexibility in approaches. Starts to be more creative despite making mistakes. Feeling overwhelmed and uncertain is common but still pursues being a doula. “Whoa, none of this is under my control, and there's a bigger bag of stuff out there than I realized.” Purpose: Directly interacts with others to support the client in getting their ideal birth. Identity statement: “I’m a doula.”
Seasoned	Theme: Knows their strengths. Can adapt what they know to different situations. Still reviews techniques and skills they have not used in a while. Feels confident some to most of the time that they can handle what comes their way as a doula. “I can use things in that birth room in ways that you can't even imagine. But if I don't have the confidence to do it, all that information in the world doesn't help me.” Purpose: Accompany clients as they go through their birth experience. Identity statement: “I am a professional doula or I work as a doula.”
Proficient	Theme: Relies more on their own experience rather than book learning in their doula work. Feels consistently confident and competent at births. Has handled a multitude of situations and developed effective communication skills with staff and clients. Is able to immediately adapt to the situation as it presents itself. “None of my perspective matters. I don't have to go home with the consequences…You have to make these really hard decisions sometimes, and your care provider has to make really hard decisions sometimes. I'm here to facilitate communication more than anything.” Purpose: Trust themselves in all aspects of interacting with a client and their family. Identity statement: “I’m a doula in every aspect of my life. I’ll never not be a doula even when I’m no longer working with families.”
Expert	Theme: Relies on intuition in problem-solving. Is adaptable in all situations including extraordinary ones. Can tell experience and skill level of other health professionals and adapts their own approach to the team. Takes confidence in their own abilities for granted. Offers tailored support without thinking about it. Labor support and doula skills seem to flow naturally without doubt. *“*What do you do in labor for pain coping?” I’m just like, “Oh dear, I don't know. Whatever pops into my head at the moment.” Purpose: Be fully open and conscious to the experience their clients are having and meet their needs no matter what happens. Identity statement: “I’ve turned my doula experience into an asset for another profession or degree (i.e., doula trainer, nurse, doula agency owner, degree in public health).”

## Discussion

### Interpreting the stages of doula development

This study demonstrates that birth doulas develop skills along a continuum with five stages of expertise: novice, advanced beginner, seasoned, proficient, and expert. While growth was influenced by the number of births attended, variation in settings and the capacity for reflection and adaptation were equally important. These findings extend the Dreyfus Model of Skill Acquisition and Benner's nursing adaptation by emphasizing that doula expertise depends as much on emotional presence and relational skill as on hands-on techniques.

A hallmark of doula development is reflective practice. Reflection and mindfulness shape how doulas adapt to new situations, turning experience into actionable knowledge (30). This iterative process of testing, modifying, and reapplying informal theories in practice closes the gap between theory and practice and makes professional growth sustainable (37).

Another key developmental transition is the shift in attribution—recognizing the doula's own role as a primary influence in the birth environment. Similar to research on expert teachers (45), who attribute classroom outcomes to the quality of teacher–student interactions, experienced doulas increasingly identified themselves as central actors shaping dynamics among clients, partners, and healthcare staff. This shift represents a move from externalized explanations of outcomes to an internalized sense of agency.

Progression from the novice to advanced beginner stages aligns with what Peña has called “pre-confident” practice, characterized by uncertainty and reliance on rules (37). As doulas gain experience, they develop individualized guidelines and begin to integrate more sophisticated emotional support strategies. Prior research has shown that doulas employ nine such strategies; in our data, early-stage practitioners most often used reassurance, encouragement, praise, and explanation, while more experienced doulas employed reframing, reinforcing, acceptance, mirroring, and debriefing. These latter strategies require greater foresight, empathy, and emotional intelligence—capacities that increase with experience.

The expert stage is marked by the emergence of intuition, a skill described in both nursing and educational literatures as the capacity to integrate conscious reasoning with tacit knowledge. Expert doulas, like expert nurses, move beyond rule-based responses to anticipate likely scenarios and flexibly shift strategies as conditions change. Intuition at this stage is not naïve guesswork but an embodied trust in accumulated experience, pattern recognition, and relational sensitivity. The expert no longer needs to refer to the rules of practice as they have themselves become the rule makers of practice (38, 41).

Taken together, these developmental patterns suggest that doula expertise is not simply a matter of attending a certain number of births, but rather a process of reflective practice, increasing self-awareness, and growing ability to anticipate and influence complex social dynamics. This highlights the importance of training and mentorship approaches that deliberately cultivate reflection, relational attunement, and situational forecasting in addition to technical skills.

### Comparison with nursing models of expertise

Recent nursing studies have continued to validate the Benner model. Healy emphasizes the role of internal engagement in developing expert practice (46). As Rosander observed in midwifery education, embodied learning and situational sensitivity grow over time, especially in unpredictable environments (47). These developments underscore how experiential, reflective, and systemic factors converge in professional growth—paralleling what was observed in doulas.

In the Dehn (30) study, nurses used a patient-centered model. Skills that were key in their development were mastering unpredictability, complexity, meaningfulness of the work, and self-reflection and awareness. These same themes were present in the excerpts from birth doulas as they grew in each stage. In a study of novice and expert nurses given patient care scenarios, novice nurses collected 49 different cues (48). They tended to be more reactive and then tried to collect cues in response to the emerging problem. The expert nurses collected 89 different cues, using a greater number and broader range of cues. These nurses were proactive in planning ahead and anticipating what would happen. For birth doulas, this shift from reaction to anticipation was seen by the time they reached the proficient stage. Expert intuition can be explained as the ability to utilize metacognitive strategies from a highly organized knowledge base to recognize patterns and then select, monitor, and modify a strategy as needed (48, 49). In their stories, the expert doulas in this study actively shifted between different possible scenarios as labor progressed and collected more cues from the person laboring and the environment.

Similar to birth doula skills, the Benner model revealed the non-clinical skills used by emergency room nurses (50). First was the ability to form compassionate connections that support their emotional availability to patients. The second cluster concerned the nurse's growing awareness that the patient needed to be protected from harm. The third cluster pertained to self-assuredness in their judgment and decision-making. The fourth cluster was communication skills. In order to flourish as emergency room nurses, they needed to become skilled in all four domains. Birth doulas need to grow in these same areas in order to succeed. These themes were represented in the findings, including awareness of patient harm.

### Systemic challenges and social context

The finding that doulas consistently described maltreatment reflects a broader reality. Maltreatment of people in labor is widespread, with an estimated 1 in 6 patients experiencing medical abuse during labor, including being yelled at and physically violated (51). It is considered a global health challenge (51–53). All doulas in the study described incidents of maltreatment, some at every birth they had attended. Doulas in the first wave of data collection described more egregious incidents of patient abuse. The last wave of doulas described maltreatment as commonplace. Patients of color are likely to experience racism in their treatment and care (54–56). One reason for having a doula is to prevent being manipulated or pushed into interventions.

All birth doulas in the study described some negative treatment (i.e., ignoring the doula, disparaging comments) from providers and clients' family members. Some experienced racism. Students who experience microaggressions have additional cognitive load and are distracted from the learning task (57). Thus, negative treatment puts an additional burden on skill development. Instead of reflecting solely on what happened at a birth, doulas go through an additional coping and debriefing process. If it also involved their client, even tangentially, that is an extra burden. Thus, skill development is a complex social process shaped by social hierarchy, culture, and unconscious bias (52).

### Strengths and limitations

The strength of this study is the large and diverse sample of 65 doulas and the 20-year time span. Interviews were lengthy and open-ended, with line-by-line coding that allowed for subtle themes to emerge. The researcher's depth of experience allowed for a detailed understanding of the connection between the inner world of doulas and outer behaviors. Multiple rigor and reflexivity strategies were implemented to involve participation from other doulas to validate emerging concepts. The limitations of this study included the following: there was only one coder; novice doula data were obtained by more experienced doulas rather than directly from this group; and efforts were made to increase the racial diversity to reflect the more current doula workforce. The context in which doula support occurs and how it is experienced may depend on race as well as other factors (i.e., ethnicity, immigrant status, and disability status). However, the actions of labor support may be a human activity that occurs similarly despite the context, making race or other socially constructed factors less influential. Determining what actions of labor support and caregiving are affected by race (or other socially constructed factors) and how they affect the process of professional development in birth doulas is a topic for future research. These efforts would be best received if they emerged from the Black doula community to encourage participation and relevance. Other areas for exploration are novice doulas and their emerging development, whether growth in these stages is linear or looping, and how particular skills progress across the stages from novice to expert.

### Implications

Effective labor support by a birth doula is a sophisticated activity deserving of compensation commensurate with the skill set of doulas. Like beginning nurses, beginning doulas are competent enough to earn a living wage with benefits. Rather than continually training new people, programs should concentrate on removing barriers to continuing birth doula work. Doula programs should address the challenges of each stage, thus encouraging greater expertise and proliferation of an experienced workforce. Seasoned, proficient, and expert doulas have much to offer as mentors and trainers to emerging doulas and deserve career pathways that recognize their expertise. Higher-level doulas are essential to any perinatal healthcare strategy that desires to improve maternal or infant outcomes; it is time they are recognized as skilled professionals.

## Conclusion

Using Benner interpretation of the Dreyfus model, this study confirms that birth doulas, like nurses, develop skills through direct client care. The process of acquiring skills and the professional contexts in which that learning occurs are comparable. Doulas and nurses face similar choices in unpredictable environments and develop professional judgment through experience. This is evident in the emergence of intuitive knowing in both expert nurses and expert doulas. However, their skill sets diverge. Nurses build clinical competencies to deliver medical care, while doulas cultivate emotional support skills that are personal, responsive, and often undervalued because they are invisible to outside observers. The skills of doulas evolve from self-reflection, adaptability, and responsiveness to client needs, rather than through observable clinical interventions (8).

This study highlights the complexity and sophistication of doula work. One-third of participants were hospital-based doulas who met their clients for the first time during labor. Yet, across all contexts, these doulas demonstrated high levels of emotional intelligence—particularly in self-awareness, relational navigation, and boundary setting. Those who remained in practice long term described the importance of emotion regulation, communication, and negotiating professional presence. They also showed significant flexibility in responding to evolving birth environments. Sustainability in doula practice depended not only on skill development but also on the ability to practice holistic self-care and to process repeated exposure to mistreatment in medical settings. Intuition emerged as a hallmark of expert-level doula work—mirroring how expert status is recognized in other fields that apply the Benner model.

## Data Availability

The datasets presented in this article are not readily available because data are available to confirm the findings. Requests to access the datasets should be directed to Amy Gilliland, amyg@fastmail.com.

## Footnotes

^1^In Dreyfus and Dreyfus and Benner's work, the third stage was “competent”. This was confusing, as a novice doula is considered competent to support families. The term that emerged from the data was “seasoned”. To be seasoned implies that one has benefitted from experience and maneuvered through challenges, a more accurate description of this stage.

^2^The use of the active verb “doulaing” to describe their activities was almost universal among participants. It encompassed both the observable tasks and the internal processes. Phrases such as “doula work”, “doula support”, or “doula practice” refer to one aspect of “doulaing”.
